# Sirtuin 1 activator alleviated lethal inflammatory injury *via* promotion of autophagic degradation of pyruvate kinase M2

**DOI:** 10.3389/fphar.2023.1092943

**Published:** 2023-04-10

**Authors:** Shuang Zhao, Yili Sun, Xicheng Wu, Yongqiang Yang, Kerui Fan, Kai Hu, Yasha Qin, Kexin Li, Ling Lin, Kun Chen, Yuhua Ma, Min Zhu, Gang Liu, Li Zhang

**Affiliations:** ^1^ Department of Pathophysiology, Basic Medical College, Chongqing Medical University, Chongqing, China; ^2^ Laboratory of Stem Cell and Tissue Engineering, Chongqing Medical University, Chongqing, China; ^3^ Medical Sciences Research Center, University-Town Hospital of Chongqing Medical University, Chongqing, China; ^4^ Xinjiang Key Laboratory of Clinical Genetic Testing and Biomedical Information, Karamay, China; ^5^ Department of Emergency and Critical Care Medicine, University-Town Hospital of Chongqing Medical University, Chongqing, China

**Keywords:** pyruvate kinase M2, sirtuin 1, autophagy, endotoxemia, deacetylase

## Abstract

Upregulation of pyruvate kinase M2 (PKM2) is critical for the orchestration of metabolism and inflammation in critical illness, while autophagic degradation is a recently revealed mechanism that counter-regulates PKM2. Accumulating evidence suggests that sirtuin 1 (SIRT1) function as a crucial regulator in autophagy. The present study investigated whether SIRT1 activator would downregulate PKM2 in lethal endotoxemia *via* promotion of its autophagic degradation. The results indicated that lethal dose of lipopolysaccharide (LPS) exposure decreased the level of SIRT1. Treatment with SRT2104, a SIRT1 activator, reversed LPS-induced downregulation of LC3B-II and upregulation of p62, which was associated with reduced level of PKM2. Activation of autophagy by rapamycin also resulted in reduction of PKM2. The decline of PKM2 in SRT2104-treated mice was accompanied with compromised inflammatory response, alleviated lung injury, suppressed elevation of blood urea nitrogen (BUN) and brain natriuretic peptide (BNP), and improved survival of the experimental animals. In addition, co-administration of 3-methyladenine, an autophagy inhibitor, or Bafilomycin A1, a lysosome inhibitor, abolished the suppressive effects of SRT2104 on PKM2 abundance, inflammatory response and multiple organ injury. Therefore, promotion of autophagic degradation of PKM2 might be a novel mechanism underlying the anti-inflammatory benefits of SIRT1 activator.

## Introduction

A growing body of evidence suggests that metabolic reprogramming would be actively involved in the orchestration of metabolic processes and inflammatory responses under various pathological circumstance including critical illness, metabolic syndrome, tumor, et al. (Mazumdar et al., 2020). Pyruvate kinase M2 (PKM2), a key enzyme in the last step of glycolysis, has been regarded as a crucial regulator in inflammatory diseases such as sepsis, asthma as well as encephalomyelitis (Yang et al., 2014; Damasceno et al., 2020; Manuel et al., 2021). PKM2 is significantly upregulated in inflammatory response, which promotes the expression of pro-inflammatory genes *via* a diverse of mechanisms (Yang et al., 2014; Palsson-McDermott et al., 2015). Previous studies have found that hypoxia inducible factor 1 (HIF-1) as well as other transcriptional factors were involved in the upregulation of PKM2 expression (Ma et al., 2015; Li et al., 2022), but the turnover of PKM2 in inflammatory response remains unknown.

Autophagy is a self-degradation process that transports cellular contents to lysosomes for degradation, which has been considered as an evolutionarily conserved adaptive response (Parmar et al., 2022). Accumulating evidence suggests that autophagy, *via* degradation of key components in inflammation, is involved in the negative regulation of inflammation and suppressed autophagy contributes to the development of lethal inflammatory injury (Lin et al., 2022; Liu et al., 2022). Recent studies have found that activation of autophagy by low-intensity pulsed ultrasound promoted the degradation of PKM2 in macrophages (Zhang et al., 2020a). In addition, autophagic degradation of PKM2 has been suggested to be involved in endothelial-to-mesenchymal transition (Gao et al., 2020). Thus, promotion of PKM2 autophagic degradation might be a novel approach to control the level of PKM2.

Acetylation as well as other post-translational modifications (PTMs) plays essential roles in the regulation of autophagy (Xu and Wan, 2022). Sirtuin 1 (SIRT1), a longevity factor, is a well-studied deacetylase that removes acetyl group from the lysine residue from acetylated proteins (Yang et al., 2022). Previous studies have found that SIRT1 functions as a crucial regulator at different stages of autophagy and activation of SIRT1 promoted autophagy (Baeken, 2023). In addition, treatment with SIRT1 activator has been suggested as an effective approach to alleviate inflammatory injury (Yang et al., 2022). Therefore, we hypothesized that activation of SIRT1 might promote autophagic degradation of PKM2, and thus attenuated lethal inflammatory injury.

Lipopolysaccharide (LPS), a major toxic component of Gram-negative bacteria, is a representative pathological factor that strongly activates of inflammatory cascade (Gorman and Golovanov, 2022). A growing body of evidence indicates that the level of LPS in serum increased, also known as endotoxemia, under a serial of pathological circumstance (Munford, 2016). In the most serious inflammatory situation, endotoxemia is involved in the development of lethal inflammation in patients with sepsis or infectious shock (Kellum et al., 2023). Systemic exposure to LPS is widely used to induce endotoxemia with lethal inflammatory injury in experimental studies (He et al., 2021; Zhang et al., 2022a). In the present study, SRT2104, a sirt1 activator (Wu et al., 2022), was administered in mice with lethal endotoxemia to investigate the pharmacological significance of SIRT1-autophagy-PKM2 pathway in inflammatory injury.

## Materials and methods

### Chemicals and reagents

Lipopolysaccharide (LPS, O55:B4, #01473370) was purchased from Sigma-Aldrich (St. Louis, MO, United States). Sirtuin 1 activator SRT2104 (#SC0272) was purchased from Beyotime Biotech (Shanghai, China). 3-methyladenine (3-MA, #HY-19312), Bafilomycin A1 (BafA1, #HY-100558) and Rapamycin (#S1842) were provided by Med Chem Express (Shanghai, China). The detection kit for blood urea nitrogen (BUN, #C013-2-1) was obtained from Nanjing Jianchen Bioengineering Institute (Nanjing, China). The dsDNA Assay Kit (#Q33263), the bicinchoninic acid (BCA) protein assay kit (#23227) and the enhanced chemiluminescence (ECL) reagents (#WP20005) were provided by Thermo Fisher Scientific (Rockford, IL, United States). The Evo M-MLV Mix Kit (#AG11728) and the SYBR Green Premix Pro Taq HS qPCR Kit (#AG11701) were purchased from Accurate Biology (Changsha, China). The enzyme linked immunosorbent assay (ELISA) kits for determination of mouse tumor necrosis factor-α (TNF-α, #EMC102a), interleukin-6 (IL-6, #EMC004.96) and monocyte chemotactic protein-1 (MCP-1, #EMC113.96) were purchased from NeoBioscience Technology Company (Shenzhen, China), the ELISA kits for C-X-C motif chemokine ligand 1 (CXCL1, #EK296/2-96), CXCL2 (#CK2142/2-96) and myeloperoxidase (MPO, #EK2133/2-96) were purchased from Multi Sciences Biotech (Hangzhou, China), and the ELISA kits for brain natriuretic peptide (BNP) was purchased from USCN Business (Wuhan, China). The antibodies for determination of AMPK (#2603), phosphorylated AMPK (p-AMPK, #2535), mTOR (#2983), p-mTOR (#5536), PKM2 (#4053) and the HRP-linked anti-rabbit (#7074) or anti-mouse (#7076) second antibodies were purchased from Cell Signaling Technology (Danvers, MA, United States). The antibodies against SIRT1(#AF0282), S6K1 (#AF0258), p-S6K1 (#AF5889), 4EBP1 (#AG 1824), p-4EBP1 (#AF5806) and LC3B (#AF5225) were provided by Beyotime Biotech (Shanghai, China). The antibodies for p62 (#18420-1-AP) and β-actin (#4ab000001) were provided by Proteintech (Wuhan, China) and 4A Biotech (Beijing, China), respectively.

### Scheme of the animal experiments

C57BL/6J mice aged 6−8 weeks with weights of 20–22 g were purchased from animal experimental center of Chongqing Medical University (Chongqing, China). The temperature of the feeding room was kept at 20−25°, and the humidity was 50%–60%. The mice were fed water and food *ad libitum*. The protocols of animal experiment were approved by the Ethics Committee of Chongqing Medical University.

Lethal endotoxemia was induced in mice with intraperitoneally injection of LPS (10 mg/kg, dissolved in normal saline). To investigate the pharmacological significance of SIRT1 activator, SRT2104 (25 mg/kg, dissolved in DMSO) was administered intraperitoneally at 30 min before LPS exposure. The experimental animals were sacrificed 8 h post LPS exposure, the serum and lung samples were collected for further experiments.

To investigate the role of autophagy in the regulation of PKM2, rapamycin (5 mg/kg, dissolved in DMSO) was administered intraperitoneally at 30 min before LPS exposure. Then, the mice were sacrificed and the lung samples were collected 8 h post LPS exposure.

To investigate whether the modulatory effects of SRT2104 depend on autophagic degradation, endotoxemic mice were injected with SRT2104 30 min before LPS exposure, and then the autophagy inhibitor 3-MA (15 mg/kg, dissolved in DMSO) or the lysosome inhibitor Bafilomycin A1 (1 mg/kg, dissolved in DMSO) was administered intraperitoneally at 30 min before SRT2104 injection. The serum and lung samples were collected 8 h post LPS exposure.

### Evaluation of the clinical status of the experimental animals

The body temperature of the mice was determined at 0 h, 2 h, 4 h, 6 h, and 8 h post LPS exposure with a mouse rectal temperature thermometer (Zhongjiao, China). The clinical status of mice was blindly scored based on the methods described previously (Weber et al., 2015; Fan et al., 2018), the clinical score of each animal is the total of the points from the categories listed in Table 1. A higher score means a worse clinical situation of the experimental animal.

**TABLE 1:** The scoring points for clinical evaluation.

Points	Coat	Activity	Respiration	Posture	Stool
1	smooth	normal	normal	moving or resting normally	normal
2	mild ruffling	moves slowly without stimulation	labored	huddled	diarrhea
3	significant ruffling	moves only with stimulation	irregular		
4		minimal movement with stimulation			

### Survival analysis

The survival of the experimental animals was recorded every 6 h for 7 days, and the survival rate of the mice was analyzed by Kaplan-Meier curve.

### Lung histopathologic examination

The left lung tissues of mice were immobilized in 4% paraformaldehyde and then encapsulated in paraffin. The samples were sectioned into 5.0 μm slices for staining with hematoxylin and eosin (H&E). Histological abnormalities of lung tissues were visualized under a light microscope (Olympus, Japan) and blindly scored according to the scoring method described previously (Fan et al., 2018). Briefly, the degree of histological abnormalities was scored on the following features: congestion, edema, inflammation, and hemorrhage with a scale of 0–4 (0, normal; 1, light; 2, moderate; 3, strong; 4, intense). The histological score is the total of the points from the four features and a higher score means more severe histological lesions.

### ELISA

The serum samples were collected 8 h post LPS exposure for the determination of TNF-α, IL-6, MCP-1, CXCL1 and CXCL2 with the ELISA kits following the manufacturer’s instructions. In addition, lung tissues were homogenized and centrifuged at 12,000 g (4°C) for 15 min. The supernatant was collected for the determination of MPO level according to the manufacture’s protocols. The OD was detected at 450 nm by microplate reader. The concentrations of these molecules were calculated based on standard curves.

### Quantitative RT-PCR analysis

Total RNA was extracted from lung tissues with using Trizol reagent (Takara, Japan) following the manufacturer’s instruction. The RNA was reversed transcription to cDNA by using an Evo M-MLV Mix Kit with gDNA Clean for qPCR (Accurate Biology). Subsequently, cDNA was amplified using the SYBR Green Premix Pro Taq HS qPCR Kit (Accurate Biology) by real-time PCR on 7,300 Real-time PCR system with the primers listed in Table 2.

**TABLE 2:** The primer sequences for qPCR.

Gene	Forward primer (5′ to 3′)	Reverse primer (5′ to 3′)
*GAPDH*	*TGT​GTC​CGT​CGT​GGA​TCT​GA*	*TTG​CTG​TTG​AAG​TCG​CAG​GAG*
*IL-6*	*GAG​GGA​TAC​CCC​CAA​CAG​ACC*	*TGC​AAT​AAC​CAC​CCC​TGA​CC*
*TNF-*α	*AAG​AGG​GAG​AGA​AGC​AAC​TAC​A*	*TGG​GTC​AGT​ATG​TGA​GAG​GAA​G*
*MCP-1*	*TGC​TGA​CCC​CAA​GAA​GGA​AT*	*TGT​GGA​AAA​GGT​AGT​GGA​TGC*
*CXCL1*	*GGC​TGG​GAT​TCA​CCT​CAA​GAA*	*GTG​GCT​ATG​ACT​TCG​GTT​TGG*
*CXCL2*	*CTC​AAC​GGA​AGA​ACC​AAA​GAG​AAA*	*CTC​AGA​CAG​CGA​GGC​ACA​T*

### Western blot analysis

Total proteins of lung tissues were extracted for western blot analysis. All the samples were lysed in RIPA buffer with protease and phosphatase inhibitors. The concentration of proteins was determined with the BCA Protein Assay kit. Protein samples were electrophoretically isolated on 6%, 7.5%, 10% or 12.5% SDS-PAGE gels and electro-transferred onto PVDF membranes, followed by blocking with 5% skim milk at room temperature for 2 h. Subsequently, the membranes were incubated overnight at 4°C with the primary antibodies against AMPK (1: 1000), p-AMPK (1: 1000), p-mTOR (1: 1000), mTOR (1: 1000), p-S6K1 (1: 1000), S6K1 (1: 1000), p-4EBP1 (1: 1000), 4EBP1 (1: 1000), p62 (1: 1000), LC3B (1: 1000), PKM2 (1: 1000) or β-Actin (1: 4000). Then, the membranes were washed and incubated with HRP-linked second antibody (1: 4000) for 2 h at room temperature. After washing, the membranes were assayed with enhanced chemiluminescence (ECL) reagent. The blots were semi-quantified by Image Lab (Bio-Rad, version 5.2).

### Statistical analysis

All data were presented as means +standard deviation (SD) and the analyses were performed by GraphPad Prism (version 8.0, San Diego, CA). Differences between groups were tested by one-way ANOVA with Tukey’s *post hoc* test. Statistical significance was considered at *p* < 0.05.

## Results

### SRT2104 activated autophagy and decreased PKM2 level

**FIGURE 1:**
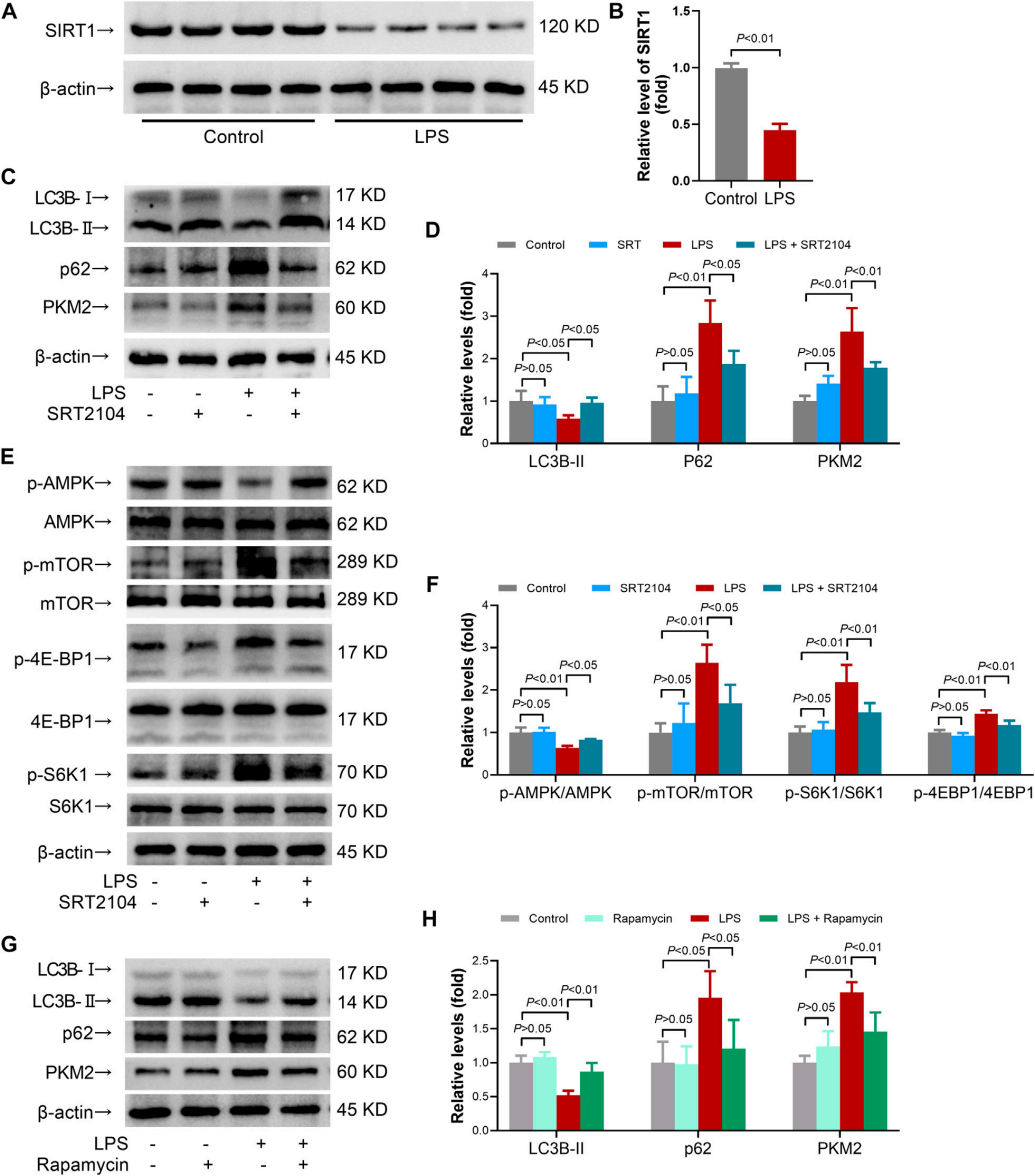
SRT2104 activator stimulated autophagy but decreased PKM2 level in LPS-insulted mice. **(A,B)** C57BL/6 mice were challenged with LPS to induce systemic inflammation. The mice were sacrificed 8 h post LPS exposure, and the lung samples were collected. **(A,B)** The protein levels of SIRT1 in lung tissues were determined; *n* = 4. **(C–F)** C57BL/6 mice were challenged with LPS to induce systemic inflammation, vehicle or SRT2104 (25 mg/kg) was administered intraperitoneally. The mice were sacrificed 8 h post LPS exposure, and the lung samples were collected. **(C,D)** The protein levels of LC3B-II, p62 and PKM2 in lung tissues were determined; **(E,F)** The phosphorylation and total protein levels of AMPK, mTOR, 4E-BP1 and S6K1 in lung tissues were determined; *n* = 4. **(G,H)** C57BL/6 mice were challenged with LPS to induce systemic inflammation, vehicle or rapamycin (5 mg/kg) was administered intraperitoneally. The mice were sacrificed 8 h post LPS exposure, the lung samples were collected and the protein levels of LC3B-II, p62 and PKM2 in lung tissues were determined, *n* = 4. Data were expressed as means +SD.

SIRT1 has been regarded as an important regulator in autophagy (Xu and Wan, 2022). The present study found that LPS-induced inflammatory is associated with reduced level of SIRT1 (Figures 1A, B). To investigate whether activation of SIRT1 could enhance autophagy and then modulate PKM2 abundance, a SIRT1 activator, SRT2104(21), was administered. A previous study has found that short term (2 weeks) or long term (>40 weeks) oral administration of SRT2104 exhibited no detectable toxic effects in experimental animals (Mercken et al., 2014). In agreement, the present study found that acute intraperitoneal administration (8 h) of SRT2104 (25 mg/kg) had little effect on body weight of the mice or the level of ALT/AST (Supplementary Figure S1), suggesting that treatment with SRT2104 might be safe in the present study.

In addition, the results indicated that LPS exposure decreased the level of LC3B-II, the most widely used molecular marker of autophagy (Baeken et al., 2020), in lung tissue, which was reversed by SRT2104 (Figures 1C, D). Treatment with SRT2104 also prevented LPS-induced elevation of p62 (Figures 1C, D). Consistently, the immunofluorescence analysis indicated that LPS suppressed the formation of LC3B-II puncta while treatment with SRT2104 reversed the suppressive effects of LPS on LC3B-II puncta (Supplementary Figure S2A). LPS exposure also resulted in elevation of p62, which was suppressed by SRT2104 (Supplementary Figure S2B). Interestingly, SRT2104 administration significantly reduced pulmonary level of PKM2 in LPS-challenged mice (Figures 1C, D). Thus, treatment with SRT2104 might activate autophagy and downregulate PKM2.

AMP-activated protein kinase (AMPK)/mammalian target of rapamycin (TOR) pathway plays central roles in the regulation of autophagy, which would be profoundly modulated by SIRT1 (Xu and Wan, 2022). The present study found that treatment with SRT2104 prevented LPS-induced dephosphorylation of AMPK, which was accompanied with suppressed phosphorylation of mTOR (Figures 1E, F). Consistently, LPS-induced phosphorylation of 4E-BP1 and S6K1, two target proteins downstream mTOR, was inhibited by SRT2104 (Figures 1E, F). Thus, activation of autophagy by SRT2104 was associated with activation of AMPK and inhibition of mTOR.

To investigate the roles of autophagy in the regulation of PKM2 in inflamed-lung tissue, rapamycin, a widely used mTOR inhibitor and autophagy inducer (Ye et al., 2022), was administered. As expected, administration of rapamycin increased the level of LC3B-II but decreased the level of p62 in LPS-insulted mice (Figures 1G, H). Treatment with rapamycin also reduced the level of PKM2 (Figures 1G, H). Thus, activation of autophagy might also induce PKM2 reduction.

### SRT2104 alleviated LPS-induced inflammatory injury

**FIGURE 2:**
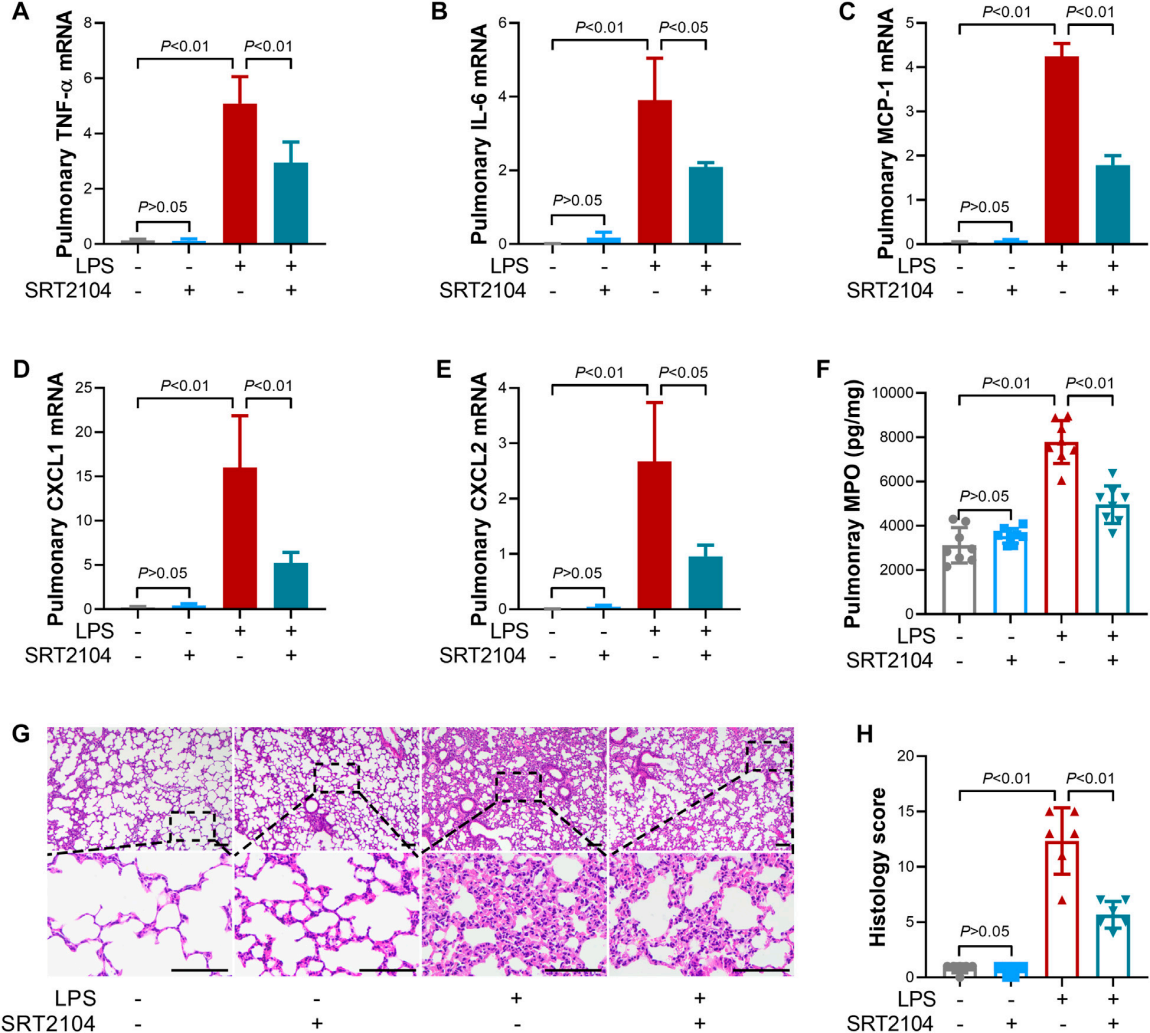
SRT2104 suppressed LPS-induced inflammatory lung injury. C57BL/6 mice were challenged with LPS to induce systemic inflammation, vehicle or SRT2104 (25 mg/kg) was administered intraperitoneally. The mice were sacrificed 8 h post LPS exposure, and the lung samples were collected. **(A–E)** The mRNA levels of **(A)** TNF-α, **(B)** IL-6, **(C)** MCP-1, **(D)** CXCL1, **(E)** CXCL2 in lung tissues were determined, *n* = 3. **(F)** The level of MPO in lung tissue was determined, *n* = 8. **(G)** HE staining were performed in lung tissues and the representative image of each group was shown (Scale bar: 100 μm); **(H)** The histological score was calculated based on the HE-stained sections; *n* = 6. Data were expressed as means +SD.

**FIGURE 3:**
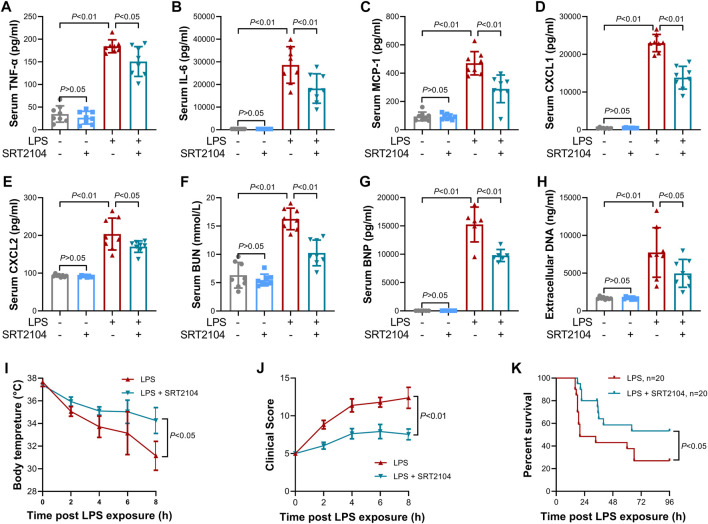
SRT2104 alleviated LPS-induced systemic inflammation. C57BL/6 mice were challenged with LPS to induce systemic inflammation, vehicle or SRT2104 (25 mg/kg) was administered intraperitoneally. The mice were sacrificed 8 h post LPS exposure, and the serum samples were collected. **(A–E)** The serum levels of **(A)** TNF-α, **(B)** IL-6, **(C)** MCP-1, **(D)** CXCL1 and **(E)** CXCL2 were determined; **(F,G)** The serum levels of **(F)** BUN and **(G)** BNP were determined; **(H)** The level of extracellular DNA in serum was determined; **(I)** The body temperature of the experimental animals was recorded; **(J)** The clinical score of the experimental animals was calculated; *n* = 8; **(K)** The survival of the experimental animals was monitored, *n* = 20. Data were expressed as means +SD.

Previous studies have suggested that PKM2 function as a crucial enhancer in inflammatory injury (Liu et al., 2021). In the present study, treatment with SRT2104 suppressed LPS-induced upregulation of both proinflammatory cytokines, such as TNF-α and IL-6, and chemokines, such as MCP-1, CXCL1 and CXCL2, in lung tissue (Figures 2A–E). In addition, SRT2104 intervention suppressed the elevation of pulmonary MPO and alleviated histological abnormalities in lung (Figures 2F, G). In agreement with the attenuated inflammatory in lung, the elevation of chemokines and cytokines in serum (Figures 3A–E), the upregulation of circulating BUN and BNP (Figures 3F, G), biomarkers for renal injury and myocardial injury (Zhang et al., 2021; Huan et al., 2022), the elevation of extracellular DNA in serum (Figure 3H), a biomarker for non-specific tissue injury (Shi et al., 2020), the decline of body temperature (Figure 3I) and the increase of clinical score (Figure 3J) were also inhibited after SRT2104 administration. Most importantly, administration of SRT2104 significantly improved the survival rate of LPS-insulted mice (Figure 3K). Therefore, decreased PKM2 is associated with alleviated inflammatory injury in endotoxemic mice with SRT2104 intervention.

### Inhibition of autophagy reversed the protective benefits of SRT2104

Autophagy has been suggested to be involved in the negative regulation of inflammation (Liu et al., 2022). Consistently, the present study found that induction of autophagy by rapamycin was accompanied with suppressed pulmonary inflammation and alleviated lung injury (Supplementary Figures S3A–F). In addition, treatment with rapamycin also blunted systemic inflammatory injury (Supplementary Figures S4A–H). Thus, reduction of PKM2 by rapamycin was associated with alleviated inflammatory injury.

**FIGURE 4:**
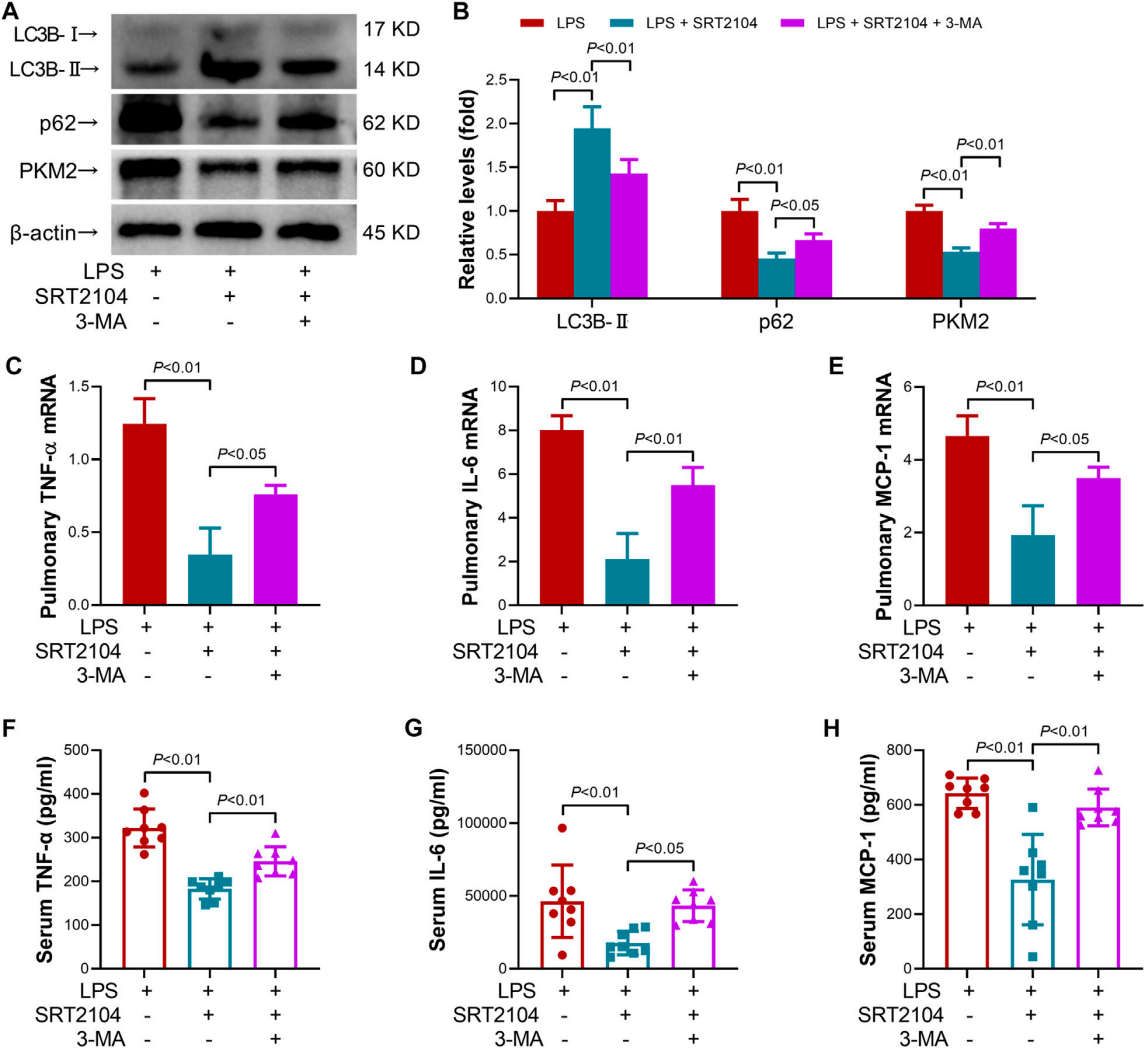
Autophagy inhibitor 3-MA reversed the suppressive effects of SRT2104 on PKM2 and inflammation. C57BL/6 mice were challenged with LPS to induce systemic inflammation, vehicle or SRT2104 (25 mg/kg) was injected intraperitoneally with or without 3-MA (15 mg/kg) co-administration. The mice were sacrificed 8 h post LPS exposure, and the lung samples as well as serum samples were collected. **(A,B)** The protein levels of LC3B-II, p62 and PKM2 in lung tissues were determined, *n* = 4. **(C–E)** The mRNA levels of **(C)** TNF-α, **(D)** IL-6 and **(E)** MCP-1 in lung tissues were determined, *n* = 3. **(F–H)** The serum levels of **(F)** TNF-α, **(G)** IL-6 and **(H)** MCP-1 were determined, *n* = 8. Data were expressed as means +SD.

**FIGURE 5:**
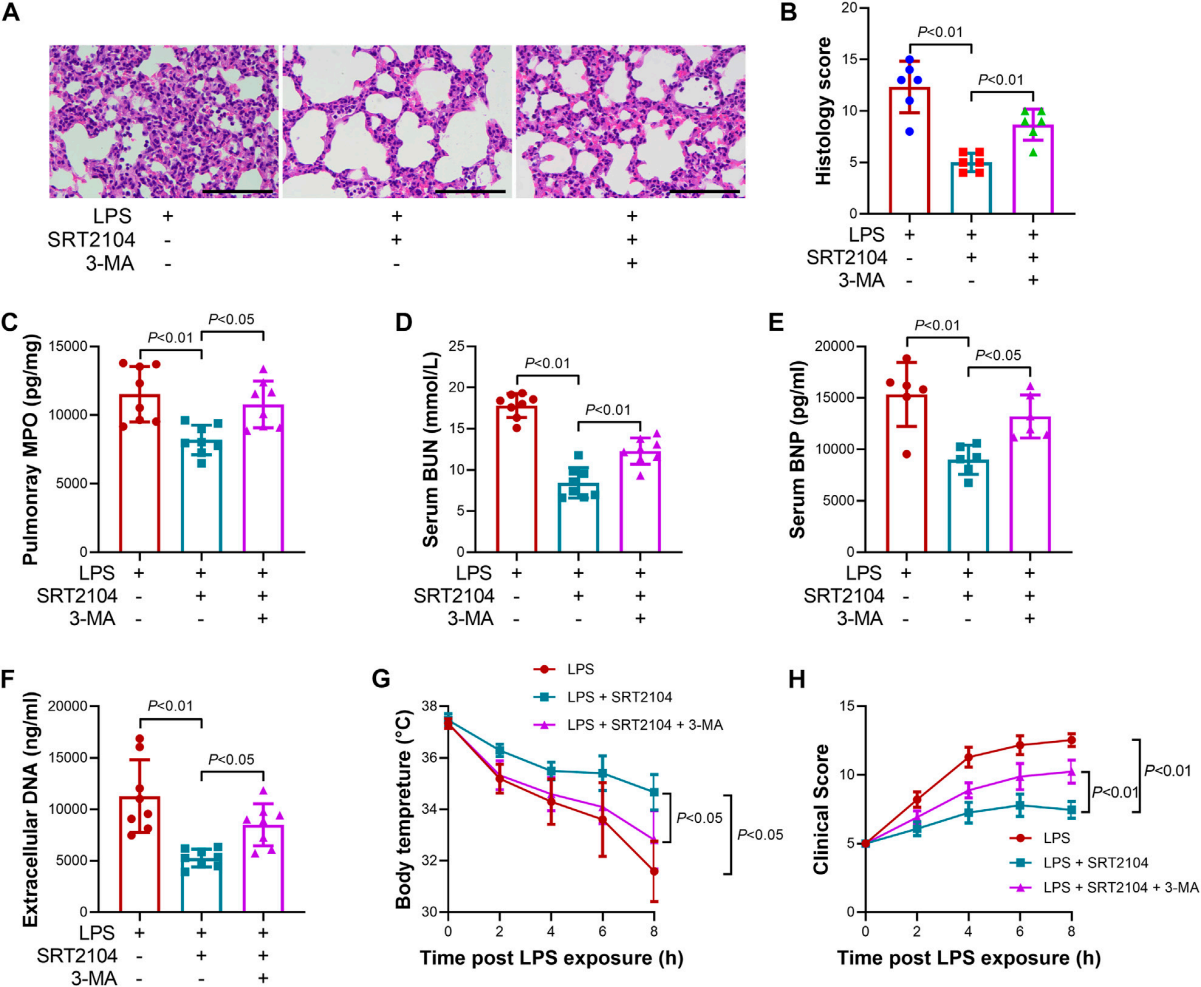
Autophagy inhibitor 3-MA reversed the beneficial effects of SRT2104 on multiple organ injury. C57BL/6 mice were challenged with LPS to induce systemic inflammation, vehicle or SRT2104 (25 mg/kg) was injected intraperitoneally with or without 3-MA (15 mg/kg) co-administration. The mice were sacrificed 8 h post LPS exposure, and the lung samples as well as serum samples were collected. **(A)** HE staining was performed in lung tissues and the representative image of each group were shown (Scale bar: 100 μm); **(B)** The histological score was calculated based on the HE-stained sections; *n* = 6. **(C)** The level of MPO in lung tissue was determined. **(D,E)** The serum levels of **(D)** BUN and **(E)** BNP were determined; **(F)** The level of extracellular DNA in serum was determined; **(G)** The body temperature of the experimental animals was recorded; **(H)** The clinical score of the experimental animals was calculated; *n* = 8. Data were expressed as means +SD.

To investigate whether the suppressive effects of ST2104 on PKM2 were autophagy-dependent, an autophagy inhibitor, 3-methyladenine (3-MA) (Zhao et al., 2022a), was co-administered with SRT2104. Administration of 3-MA prevented SRT2104-induced upregulation of LC3B-II and downregulation of p62 and PKM2 (Figures 4A, B). In addition, the suppressive effects of SRT2104 on pro-inflammatory cytokines expression were also reversed by 3-MA (Figures 4C–H). Consistently, the alleviated histological lesions in lung tissue (Figures 5A, B), the downregulation of pulmonary MPO (Figure 5C), the decreased level of BUN, BNP and extracellular DNA in serum (Figures 5D–F), the suppressed decline of body temperature (Figure 5G) and the reduced clinical score (Figure 5H) in SRT2104-treated group were reversed by 3-MA. Thus, the downregulation of PKM2 by SRT2104 might result from autophagy-dependent degradation of PKM2.

### Lysosome inhibition reversed the protective benefits of SRT2104

**FIGURE 6:**
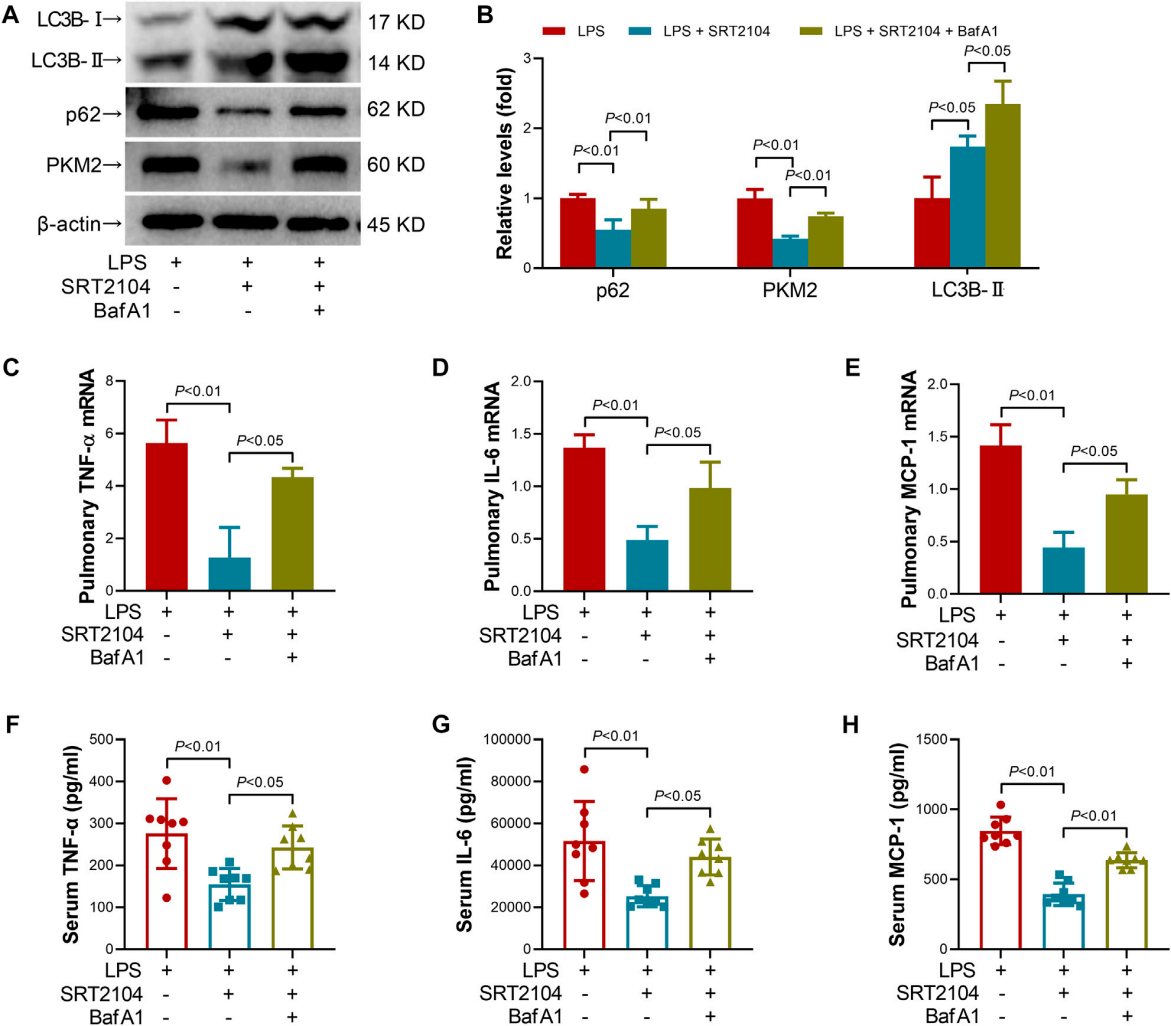
Lysosome inhibitor Bafilomycin A1 (BafA1) reversed the suppressive effects of SRT2104 on PKM2 and inflammation. C57BL/6 mice were challenged with LPS to induce systemic inflammation, vehicle or SRT2104 (25 mg/kg) was injected intraperitoneally with or without BafA1 (1 mg/kg) co-administration. The mice were sacrificed 8 h post LPS exposure, and the lung samples as well as serum samples were collected. **(A,B)** The protein levels of LC3B-II, p62 and PKM2 in lung tissues were determined, *n* = 4. **(C–E)** The mRNA levels of **(C)** TNF-α, **(D)** IL-6 and **(E)** MCP-1 in lung tissues were determined, *n* = 3. **(F–H)** The serum levels of **(F)** TNF-α, **(G)** IL-6 and **(H)** MCP-1 were determined, n = 8. Data were expressed as means +SD.

**FIGURE 7:**
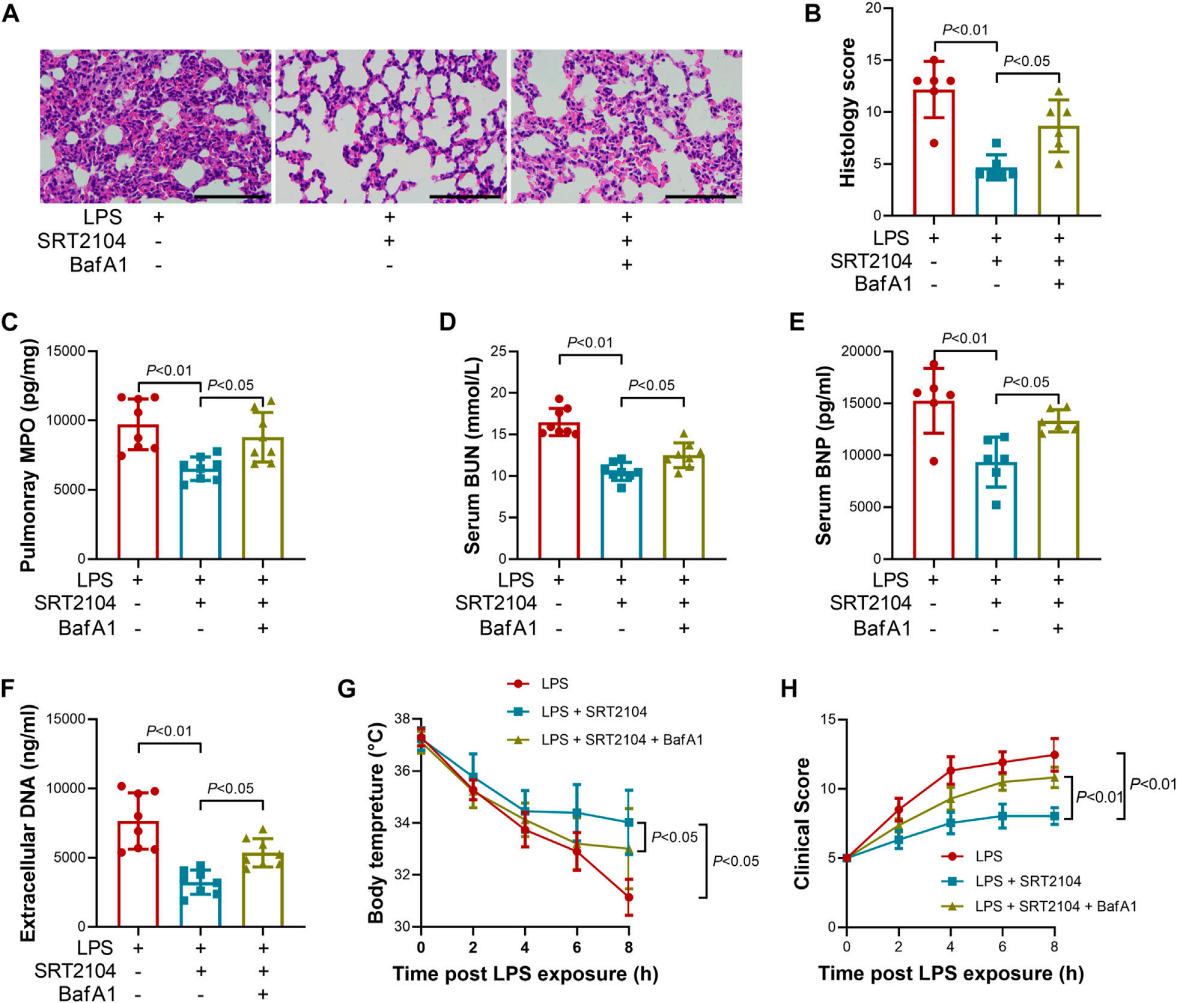
Lysosome inhibitor Bafilomycin A1 (BafA1) reversed the beneficial effects of SRT2104 on multiple organ injury. C57BL/6 mice were challenged with LPS to induce systemic inflammation, vehicle or SRT2104 (25 mg/kg) was injected intraperitoneally with or without BafA1 (1 mg/kg) co-administration. The mice were sacrificed 8 h post LPS exposure, and the lung samples as well as serum samples were collected. **(A)** HE staining was performed in lung tissues and the representative image of each group was shown (Scale bar: 100 μm); **(B)** The histological score was calculated based on the HE-stained sections; *n* = 6. **(C)** The level of MPO in lung tissue was determined; **(D,E)** The serum levels of **(D)** BUN and **(E)** BNP were determined; **(F)** The level of extracellular DNA in serum was determined; **(G)** The body temperature of the experimental animals was recorded; **(H)** The clinical score of the experimental animals was calculated; *n* = 8. Data were expressed as means +SD.

Lysosome is responsible for the degradation of cellular cargo in autophagy (Yim and Mizushima, 2020). To further confirm that the suppressive effects of SRT2104 on PKM2 depend on autophagic degradation, a lysosome inhibitor, Bafilomycin A1 (BafA1) (Baeken et al., 2020), was co-administered with SRT2104. Similarly, treatment with BafA1, prevented the reduction of LC3B-II, p62 and PKM2 in SRT2104-treated experimental animals (Figures 6A, B), which was associated with elevated expression of pro-inflammatory cytokines (Figures 6C–H). In addition, the beneficial effects of SRT2104 on lung injury as well as other organs injury were also reversed after BafA1 administration (Figures 7A–H). Thus, lysosome is responsible for the promotive effects of SRT2104 on PKM2 degradation.

## Discussion

Autophagy is emerging as a crucial regulatory in inflammation (Liu et al., 2022). Previous studies have found that autophagic degradation of inflammation-related proteins plays crucial roles in the negative regulation of inflammatory cascade (Zhang et al., 2020a; Lin et al., 2022). In the present study, LPS-induced upregulation of PKM2 was associated with suppressed autophagy, while induction of autophagy by rapamycin decreased the level of PKM2 and alleviated lethal inflammation, suggesting that autophagy is crucial for the negative regulation of PKM2 in critical illness. Therefore, autophagic degradation of PKM2, a metabolic enzyme, might be another crucial mechanism for the control of excessive inflammation by autophagy.

The upregulation of PKM2 is a critical molecular event for the exacerbation of inflammatory injury (Damasceno et al., 2020; Dhanesha et al., 2022; Doddapattar et al., 2022). Previous studies have found that deletion of PKM2 resulted in suppressed inflammatory response and beneficial outcomes in experimental animals with autoimmune encephalomyelitis, ischemic stroke as well as acute lung injury (Damasceno et al., 2020; Dhanesha et al., 2022; Sun et al., 2022). Interestingly, a growing body of evidence suggests that the induction of inflammatory injury is usually associated with suppressed autophagy (Fan et al., 2018), which might result in reduced degradation of PKM2 and then the accumulation of PKM2 in inflamed tissues. Therefore, in addition to enhanced transcription (Zhong et al., 2019), compromised autophagic degradation of PKM2 might also contribute to the upregulation of PKM2 under inflammatory circumstance, and promotion of autophagic degradation of PKM2 might be a promising strategy for the pharmacological control of inflammatory injury.

Autophagy is regulated by PTMs such as acetylation (Xu and Wan, 2022), and SIRT1 is a representative deacetylase that profoundly involved in the regulation of autophagy (Baeken, 2023). Previous studies have revealed that SIRT1 modulates autophagy at transcriptional level *via* deacetylating Forkhead Box O1 (FOXO1) and Forkhead Box O3 (FOXO3) (Hariharan et al., 2010; Dusabimana et al., 2019). In addition, SIRT1 directly deacetylates the key regulators of autophagy, such as Beclin 1 and ATG5, which promotes the formation of autophagosome (Lee et al., 2008; Sun et al., 2015). Most interestingly, SIRT1 directly deacetylates LC3-I, which is necessary for the formation of LC3-II and then the elongation of autophagosome (Huang et al., 2015). In the present study, LPS exposure induced the reduction of SIRT1, which might contribute to the suppressed autophagy in inflammation. In addition, treatment with SIRT1 activator SRT2104 alleviated inflammatory injury, enhanced autophagy, and reduced the level of PKM2, which were reversed by autophagy inhibitor or lysosome inhibitor. Thus, the beneficial effects of SRT2104 in inflammatory injury might depend on, at least partially, the activation of autophagic program and degradation of PKM2.

Additionally, a previous study has found that PKM2 is acetylated on lysine 305 and this modification promotes the autophagic degradation of PKM2 (Lv et al., 2011). Another study has reported that PKM2 is acetylated on lysine 433, which modulates the nuclear localization of PKM2 (Lv et al., 2013), but this modification does not affect PKM2 protein stability (Lei et al., 2022). In addition, PKM2 is acetylated on lysine 62 and lysine 66, but there is no evidence that acetylation on these residues modulates the stability of PKM2 (Zhang et al., 2020b; Zhang et al., 2022b). Thus, acetylation of PKM2 on these published lysine residues, including lysine 62, 66, 305 and 433, might not be responsible for the reduction of PKM2 in SIRT1 activator-treated group because none of these acetylation modifications is associated with suppression of PKM2 degradation. Several deacetylases, such as SIRT2, SIRT3, SIRT6 and histone deacetylase 8 (HDAC8), have been found to be involved in the deacetylation of PKM2 (Bhardwaj and Das, 2016; Park et al., 2016; Zhang et al., 2020b; Zhao et al., 2022b), but whether SIRT1 also directly interacts with and deacetylates PKM2 remains to be further investigated.

The anti-inflammatory properties of SIRT1 have been well documented (Xie et al., 2013; Yang et al., 2022). Previous studies have found that SIRT1 suppressed inflammatory response *via* deacetylate several inflammation-related transcriptional factors, such as nuclear factor kappa B (NF-κB), activator protein 1 (AP-1) and HIF-1, and then alters their transcriptional activity and the subsequent expression of inflammatory genes (Yang et al., 2017; Chen et al., 2022; Fu et al., 2022). However, the present study found that SIRT1 stimulates autophagic degradation of PKM2, a crucial promotor of inflammation (Liu et al., 2021), which results in reduction of PKM2 and attenuation of inflammation. To the best of our knowledge, the present study revealed a novel mechanism responsible for the anti-inflammatory benefits of SIRT1.

Although SRT2104 has been widely used as a selective SIRT1 activator both in experimental studies and clinical trials (Sands et al., 2016; Gao et al., 2021), its potential off-target effects could not be completely excluded because a previous study has reported that SRT1720, another well-studied potent SIRT1 activator, might not act as a direct activator of SIRT1 (Pacholec et al., 2010). Therefore, the roles of SIRT1 and acetylation modification in the regulation of autophagic degradation of PKM2 requires more detailed investigation. In addition, the autophagosome and lysosome are regulated by a serial of signal pathways, transcriptional factors and autophagy-related proteins, the crucial protein targets responsible for the stimulatory effects of SRT2104/SIRT1 on autophagic degradation of PKM2 also remain to be further investigated.

**FIGURE 8:**
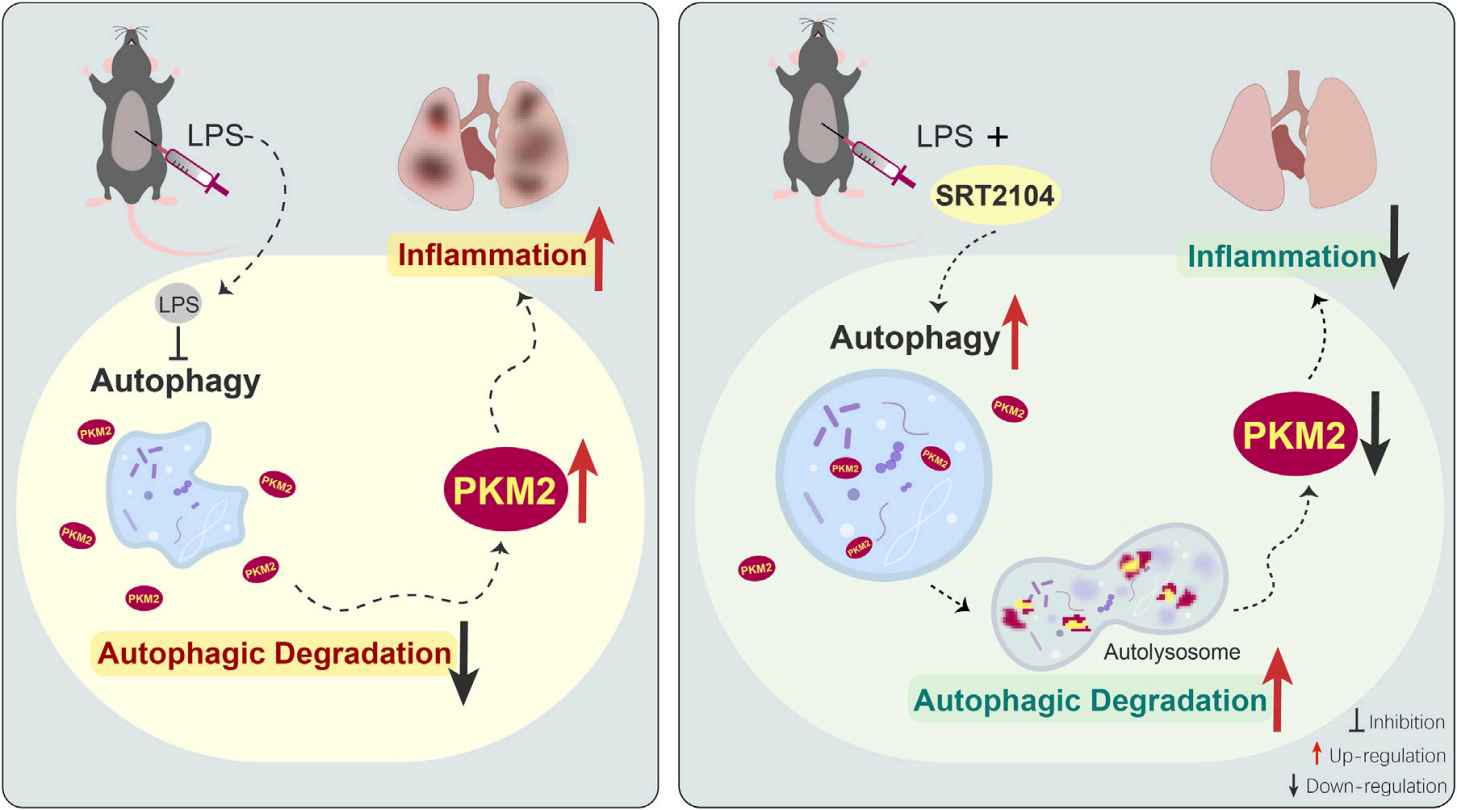
The schematic diagram of the mechanisms underlying the anti-inflammatory benefits of SRT2104 in the present study. LPS exposure suppresses autophagy, which results in compromised autophagic degradation of PKM2 and the subsequent upregulation of PKM2, amplification of inflammation and exacerbation of inflammatory injury. Administration of SRT2104 boosts autophagy, and then promotes the degradation of PKM2 and the alleviation of inflammatory injury.

Taken together, the present study found that the upregulation of PKM2 in lethal inflammation was counter-regulated by autophagic degradation, while treatment with SIRT1 activator SRT2104 stimulated autophagic degradation of PKM2 and dampened lethal inflammatory injury (Figure 8). Although the underlying mechanisms remain to be further investigated, the present study revealed a novel approach to decrease the abundance of PKM2 and weaken the orchestration of metabolism and inflammation in critical illness.

## Data Availability

The original contributions presented in the study are included in the article/Supplementary Material, further inquiries can be directed to the corresponding authors.

## References

[B1] Baeken M. W. (2023). Sirtuins and their influence on autophagy. J. Cell. Biochem. 10.1002/jcb.30377 36745668

[B2] Baeken M. W. Weckmann K. Diefenthaler P. Schulte J. Yusifli K. Moosmann B. (2020). Novel insights into the cellular localization and regulation of the autophagosomal proteins LC3A, LC3B and LC3C. Cells 9 (10), 2315. 10.3390/cells9102315 33081014 PMC7603224

[B3] Bhardwaj A. Das S. (2016). SIRT6 deacetylates PKM2 to suppress its nuclear localization and oncogenic functions. Proc. Natl. Acad. Sci. U. S. A. 113 (5), E538–E547. 10.1073/pnas.1520045113 26787900 PMC4747762

[B4] Chen Q. Cui K. Zhao Z. Xu X. Liu Y. Shen Y. (2022). LPS stimulation stabilizes HIF-1α by enhancing HIF-1α acetylation via the PARP1-SIRT1 and ACLY-Tip60 pathways in macrophages. FASEB J. 36 (7), e22418. 10.1096/fj.202200256R 35713568

[B5] Damasceno L. E. A. Prado D. S. Veras F. P. Fonseca M. M. Toller-Kawahisa J. E. Rosa M. H. (2020). PKM2 promotes Th17 cell differentiation and autoimmune inflammation by fine-tuning STAT3 activation. J. Exp. Med. 217 (10), e20190613. 10.1084/jem.20190613 32697823 PMC7537396

[B6] Dhanesha N. Patel R. B. Doddapattar P. Ghatge M. Flora G. D. Jain M. (2022). PKM2 promotes neutrophil activation and cerebral thromboinflammation: Therapeutic implications for ischemic stroke. Blood 139 (8), 1234–1245. 10.1182/blood.2021012322 34529778 PMC8874361

[B7] Doddapattar P. Dev R. Ghatge M. Patel R. B. Jain M. Dhanesha N. (2022). Myeloid cell PKM2 deletion enhances efferocytosis and reduces atherosclerosis. Circ. Res. 130 (9), 1289–1305. 10.1161/CIRCRESAHA.121.320704 35400205 PMC9050913

[B8] Dusabimana T. Kim S. R. Kim H. J. Park S. W. Kim H. (2019). Nobiletin ameliorates hepatic ischemia and reperfusion injury through the activation of SIRT-1/FOXO3a-mediated autophagy and mitochondrial biogenesis. Exp. Mol. Med. 51 (4), 1–16. 10.1038/s12276-019-0245-z PMC6486618 31028246

[B9] Fan K. Lin L. Ai Q. Wan J. Dai J. Liu G. (2018). Lipopolysaccharide-induced dephosphorylation of AMPK-activated protein kinase potentiates inflammatory injury via repression of ULK1-dependent autophagy. Front. Immunol. 9, 1464. 10.3389/fimmu.2018.01464 29988556 PMC6026648

[B10] Fu Y. Wang Y. Liu Y. Tang C. Cai J. Chen G. (2022). p53/sirtuin 1/NF-κB signaling Axis in chronic inflammation and maladaptive kidney repair after cisplatin nephrotoxicity. Front. Immunol. 13, 925738. 10.3389/fimmu.2022.925738 35874713 PMC9301469

[B11] Gao J. Chen Q. Q. Huang Y. Li K. H. Geng X. J. Wang T. (2021). Design, synthesis and pharmacological evaluation of naphthofuran derivatives as potent SIRT1 activators. Front. Pharmacol. 12, 653233. 10.3389/fphar.2021.653233 33995069 PMC8113817

[B12] Gao J. Wei T. Huang C. Sun M. Shen W. (2020). Sirtuin 3 governs autophagy-dependent glycolysis during Angiotensin II-induced endothelial-to-mesenchymal transition. FASEB J. 34 (12), 16645–16661. 10.1096/fj.202001494R 33131100

[B13] Gorman A. Golovanov A. P. (2022). Lipopolysaccharide structure and the phenomenon of low endotoxin recovery. Eur. J. Pharm. Biopharm. 180, 289–307. 10.1016/j.ejpb.2022.10.006 36272656

[B14] Hariharan N. Maejima Y. Nakae J. Paik J. Depinho R. A. Sadoshima J. (2010). Deacetylation of FoxO by Sirt1 plays an essential role in mediating starvation-induced autophagy in cardiac myocytes. Circ. Res. 107 (12), 1470–1482. 10.1161/CIRCRESAHA.110.227371 20947830 PMC3011986

[B15] He X. Dong K. Shen J. Hu G. Liu J. Kang X. (2021). Deficiency of the novel high mobility group protein HMGXB4 protects against systemic inflammation-induced endotoxemia in mice. Proc. Natl. Acad. Sci. U. S. A. 118 (7), e2021862118. 10.1073/pnas.2021862118 33563757 PMC7896282

[B16] Huan G. Tao Y. Yu W. Jinghui Z. Yueming Z. Yanqing S. (2022). Egg white protein hydrolysate ameliorated sepsis-induced inflammatory injuries in kidney and liver based on metabolomics analysis. Biomed. Pharmacother. 153, 113442. 10.1016/j.biopha.2022.113442 36076557

[B17] Huang R. Xu Y. Wan W. Shou X. Qian J. You Z. (2015). Deacetylation of nuclear LC3 drives autophagy initiation under starvation. Mol. Cell. 57 (3), 456–466. 10.1016/j.molcel.2014.12.013 25601754

[B18] Kellum J. A. Foster D. Walker P. M. (2023). Endotoxemic shock: A molecular phenotype in sepsis. Nephron 147 (1), 17–20. 10.1159/000525548 35790144

[B19] Lee I. H. Cao L. Mostoslavsky R. Lombard D. B. Liu J. Bruns N. E. (2008). A role for the NAD-dependent deacetylase Sirt1 in the regulation of autophagy. Proc. Natl. Acad. Sci. U. S. A. 105 (9), 3374–3379. 10.1073/pnas.0712145105 18296641 PMC2265142

[B20] Lei H. Yang L. Wang Y. Zou Z. Liu M. Xu H. (2022). JOSD2 regulates PKM2 nuclear translocation and reduces acute myeloid leukemia progression. Exp. Hematol. Oncol. 11 (1), 42. 10.1186/s40164-022-00295-w 35836282 PMC9281007

[B21] Li Z. Geng M. Ye X. Ji Y. Li Y. Zhang X. (2022). IRF7 inhibits the Warburg effect via transcriptional suppression of PKM2 in osteosarcoma. Int. J. Biol. Sci. 18 (1), 30–42. 10.7150/ijbs.65255 34975316 PMC8692136

[B22] Lin Y. Li Z. Wang Y. Tian T. Jia P. Ye Y. (2022). CCDC50 suppresses NLRP3 inflammasome activity by mediating autophagic degradation of NLRP3. EMBO Rep. 23 (5), e54453. 10.15252/embr.202154453 35343634 PMC9066065

[B23] Liu C. Xiao K. Xie L. (2022). Progress in preclinical studies of macrophage autophagy in the regulation of ALI/ARDS. Front. Immunol. 13, 922702. 10.3389/fimmu.2022.922702 36059534 PMC9433910

[B24] Liu Z. Le Y. Chen H. Zhu J. Lu D. (2021). Role of PKM2-mediated immunometabolic reprogramming on development of cytokine storm. Front. Immunol. 12, 748573. 10.3389/fimmu.2021.748573 34759927 PMC8572858

[B25] Lv L. Li D. Zhao D. Lin R. Chu Y. Zhang H. (2011). Acetylation targets the M2 isoform of pyruvate kinase for degradation through chaperone-mediated autophagy and promotes tumor growth. Mol. Cell. 42 (6), 719–730. 10.1016/j.molcel.2011.04.025 21700219 PMC4879880

[B26] Lv L. Xu Y. P. Zhao D. Li F. L. Wang W. Sasaki N. (2013). Mitogenic and oncogenic stimulation of K433 acetylation promotes PKM2 protein kinase activity and nuclear localization. Mol. Cell. 52 (3), 340–352. 10.1016/j.molcel.2013.09.004 24120661 PMC4183148

[B27] Ma T. Patel H. Babapoor-Farrokhran S. Franklin R. Semenza G. L. Sodhi A. (2015). KSHV induces aerobic glycolysis and angiogenesis through HIF-1-dependent upregulation of pyruvate kinase 2 in Kaposi's sarcoma. Angiogenesis 18 (4), 477–488. 10.1007/s10456-015-9475-4 26092770 PMC4659376

[B28] Manuel A. M. van de Wetering C. MacPherson M. Erickson C. Murray C. Aboushousha R. (2021). Dysregulation of pyruvate kinase M2 promotes inflammation in a mouse model of obese allergic asthma. Am. J. Respir. Cell. Mol. Biol. 64 (6), 709–721. 10.1165/rcmb.2020-0512OC 33662229 PMC8456891

[B29] Mazumdar C. Driggers E. M. Turka L. A. (2020). The untapped opportunity and challenge of immunometabolism: A new paradigm for drug discovery. Cell. Metab. 31 (1), 26–34. 10.1016/j.cmet.2019.11.014 31839485

[B30] Mercken E. M. Mitchell S. J. Martin-Montalvo A. Minor R. K. Almeida M. Gomes A. P. (2014). SRT2104 extends survival of male mice on a standard diet and preserves bone and muscle mass. Aging Cell. 13 (5), 787–796. 10.1111/acel.12220 24931715 PMC4172519

[B31] Munford R. S. (2016). Endotoxemia-menace, marker, or mistake? J. Leukoc. Biol. 100 (4), 687–698. 10.1189/jlb.3RU0316-151R 27418356 PMC5014740

[B32] Pacholec M. Bleasdale J. E. Chrunyk B. Cunningham D. Flynn D. Garofalo R. S. (2010). SRT1720, SRT2183, SRT1460, and resveratrol are not direct activators of SIRT1. J. Biol. Chem. 285 (11), 8340–8351. 10.1074/jbc.M109.088682 20061378 PMC2832984

[B33] Palsson-McDermott E. M. Curtis A. M. Goel G. Lauterbach M. A. Sheedy F. J. Gleeson L. E. (2015). Pyruvate kinase M2 regulates Hif-1α activity and IL-1β induction and is a critical determinant of the warburg effect in LPS-activated macrophages. Cell. Metab. 21 (1), 65–80. 10.1016/j.cmet.2014.12.005 25565206 PMC5198835

[B34] Park S. H. Ozden O. Liu G. Song H. Y. Zhu Y. Yan Y. (2016). SIRT2-Mediated deacetylation and tetramerization of pyruvate kinase directs glycolysis and tumor growth. Cancer Res. 76 (13), 3802–3812. 10.1158/0008-5472.CAN-15-2498 27197174 PMC4930699

[B35] Parmar U. M. Jalgaonkar M. P. Kulkarni Y. A. Oza M. J. (2022). Autophagy-nutrient sensing pathways in diabetic complications. Pharmacol. Res. 184, 106408. 10.1016/j.phrs.2022.106408 35988870

[B36] Sands B. E. Joshi S. Haddad J. Freudenberg J. M. Oommen D. E. Hoffmann E. (2016). Assessing colonic exposure, safety, and clinical activity of SRT2104, a novel oral SIRT1 activator, in patients with mild to moderate ulcerative colitis. Inflamm. Bowel Dis. 22 (3), 607–614. 10.1097/MIB.0000000000000597 26595549 PMC4885523

[B37] Shi C. Yang L. Braun A. Anders H. J. (2020). Extracellular DNA-A danger signal triggering immunothrombosis. Front. Immunol. 11, 568513. 10.3389/fimmu.2020.568513 33117353 PMC7575749

[B38] Sun T. Li X. Zhang P. Chen W. D. Zhang H. L. Li D. D. (2015). Acetylation of Beclin 1 inhibits autophagosome maturation and promotes tumour growth. Nat. Commun. 6, 7215. 10.1038/ncomms8215 26008601 PMC4455096

[B39] Sun X. Shi F. Wang W. Wu Y. Qu S. Li J. (2022). Myeloid-specific pyruvate-kinase-type-M2-deficient mice are resistant to acute lung injury. Biomedicines 10 (5), 1193. 10.3390/biomedicines10051193 35625931 PMC9138865

[B40] Weber G. F. Chousterman B. G. He S. Fenn A. M. Nairz M. Anzai A. (2015). Interleukin-3 amplifies acute inflammation and is a potential therapeutic target in sepsis. Science 347 (6227), 1260–1265. 10.1126/science.aaa4268 25766237 PMC4376966

[B41] Wu X. Ren Y. Wen Y. Lu S. Li H. Yu H. (2022). Deacetylation of ZKSCAN3 by SIRT1 induces autophagy and protects SN4741 cells against MPP(+)-induced oxidative stress. Free Radic. Biol. Med. 181, 82–97. 10.1016/j.freeradbiomed.2022.02.001 35124181

[B42] Xie J. Zhang X. Zhang L. (2013). Negative regulation of inflammation by SIRT1. Pharmacol. Res. 67 (1), 60–67. 10.1016/j.phrs.2012.10.010 23098819

[B43] Xu Y. Wan W. (2022). Acetylation in the regulation of autophagy. Autophagy 19, 379–387. 10.1080/15548627.2022.2062112 35435793 PMC9851266

[B44] Yang C. M. Chen Y. W. Chi P. L. Lin C. C. Hsiao L. D. (2017). Resveratrol inhibits BK-induced COX-2 transcription by suppressing acetylation of AP-1 and NF-κB in human rheumatoid arthritis synovial fibroblasts. Biochem. Pharmacol. 132, 77–91. 10.1016/j.bcp.2017.03.003 28288820

[B45] Yang L. Xie M. Yang M. Yu Y. Zhu S. Hou W. (2014). PKM2 regulates the Warburg effect and promotes HMGB1 release in sepsis. Nat. Commun. 5, 4436. 10.1038/ncomms5436 25019241 PMC4104986

[B46] Yang Y. Liu Y. Wang Y. Chao Y. Zhang J. Jia Y. (2022). Regulation of SIRT1 and its roles in inflammation. Front. Immunol. 13, 831168. 10.3389/fimmu.2022.831168 35359990 PMC8962665

[B47] Ye H. Chen C. Wu H. Zheng K. Martin-Adrados B. Caparros E. (2022). Genetic and pharmacological inhibition of XBP1 protects against APAP hepatotoxicity through the activation of autophagy. Cell. Death Dis. 13 (2), 143. 10.1038/s41419-022-04580-8 35145060 PMC8831621

[B48] Yim W. W. Mizushima N. (2020). Lysosome biology in autophagy. Cell. Discov. 6, 6. 10.1038/s41421-020-0141-7 32047650 PMC7010707

[B49] Zhang B. Chen H. Ouyang J. Xie Y. Chen L. Tan Q. (2020). SQSTM1-dependent autophagic degradation of PKM2 inhibits the production of mature IL1B/IL-1β and contributes to LIPUS-mediated anti-inflammatory effect. Autophagy 16 (7), 1262–1278. 10.1080/15548627.2019.1664705 31500508 PMC7469634

[B50] Zhang L. Han B. Liu H. Wang J. Feng X. Sun W. (2021). Circular RNA circACSL1 aggravated myocardial inflammation and myocardial injury by sponging miR-8055 and regulating MAPK14 expression. Cell. Death Dis. 12 (5), 487. 10.1038/s41419-021-03777-7 33986259 PMC8119943

[B51] Zhang R. Shen M. Wu C. Chen Y. Lu J. Li J. (2020). HDAC8-dependent deacetylation of PKM2 directs nuclear localization and glycolysis to promote proliferation in hepatocellular carcinoma. Cell. Death Dis. 11 (12), 1036. 10.1038/s41419-020-03212-3 33279948 PMC7719180

[B52] Zhang W. Guo X. Ren J. Chen Y. Wang J. Gao A. (2022). GCN5-mediated PKM2 acetylation participates in benzene-induced hematotoxicity through regulating glycolysis and inflammation via p-Stat3/IL17A axis. Environ. Pollut. 295, 118708. 10.1016/j.envpol.2021.118708 34929209

[B53] Zhang X. Chen C. Ling C. Luo S. Xiong Z. Liu X. (2022). EGFR tyrosine kinase activity and Rab GTPases coordinate EGFR trafficking to regulate macrophage activation in sepsis. Cell. Death Dis. 13 (11), 934. 10.1038/s41419-022-05370-y 36344490 PMC9640671

[B54] Zhao J. Wang G. Han K. Wang Y. Wang L. Gao J. (2022). Mitochondrial PKM2 deacetylation by procyanidin B2-induced SIRT3 upregulation alleviates lung ischemia/reperfusion injury. Cell. Death Dis. 13 (7), 594. 10.1038/s41419-022-05051-w 35821123 PMC9276754

[B55] Zhao X. Huang W. Shi Y. Guo J. Xiao H. Ji N. (2022). PLAAT1 inhibits type I interferon response via degradation of IRF3 and IRF7 in Zebrafish. Front. Immunol. 13, 979919. 10.3389/fimmu.2022.979919 36172355 PMC9510373

[B56] Zhong W. J. Yang H. H. Guan X. X. Xiong J. B. Sun C. C. Zhang C. Y. (2019). Inhibition of glycolysis alleviates lipopolysaccharide-induced acute lung injury in a mouse model. J. Cell. Physiol. 234 (4), 4641–4654. 10.1002/jcp.27261 30256406

