# Supplementary material for: Sirtuin 1 activator alleviated lethal inflammatory injury via promotion of autophagic degradation of pyruvate kinase M2
Source: Front Pharmacol. 2023 Apr 10;14:1092943. doi: 10.3389/fphar.2023.1092943 (PMC10123272; doi:10.3389/fphar.2023.1092943)
Supplement: Supplementary file 1 [file DataSheet1.PDF]

Supplementary methods

Determination of aminotransferase

The level of aminotransferase in serum were determined with the alanine aminotransferase (ALT, #C009-1-1), aspartate aminotransferase (AST, #C010-1-1) assay kits produced by Nanjing Jianchen Bioengineering Institute (Nanjing, China). According to the manufacturer's instruction, serum samples and the substrate solution were incubated at 37 ◦C for 30 min. Then, phenylhydrazine was added and incubated at 37 ◦C for 20 min, and finally, NaOH solution was added to stop the reaction. The OD was measured with a microplate reader (Thermo Scientific) at 505 nm, and the activities of ALT/AST were calculated based on the standard curves.

Immunofluorescence

The lung sections were incubated with blocking buffer for 60 min and then incubated with the primary antibodies against LC3 (1: 100) or p62 (1: 100) overnight at 4°C. The slices were washed with PBS and incubated with Alexa 488-conjugated anti-rabbit secondary antibody (1: 400) at room temperature for 2 h. After washing with PBS, the slices were incubated with DAPI. Finally, the images were captured under a fluorescence microscope (Olympus, Japan).

Supplementary figures and figure legends

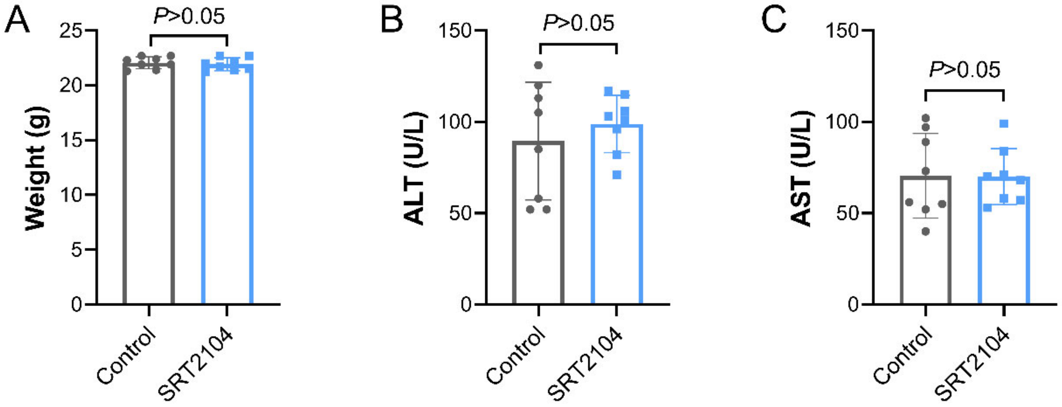

Supplementary figure 1. SRT2104 administration had little effect on body weight or serum transaminases. C57BL/6 mice were intraperitoneally injected with vehicle or SRT2104 (25 mg/kg), the mice were sacrificed 8 h later, (A) the body weight was analyzed, the serum samples were collected and the level of (B) ALT and (C) AST were determined. Data were expressed as means + SD, n=8.

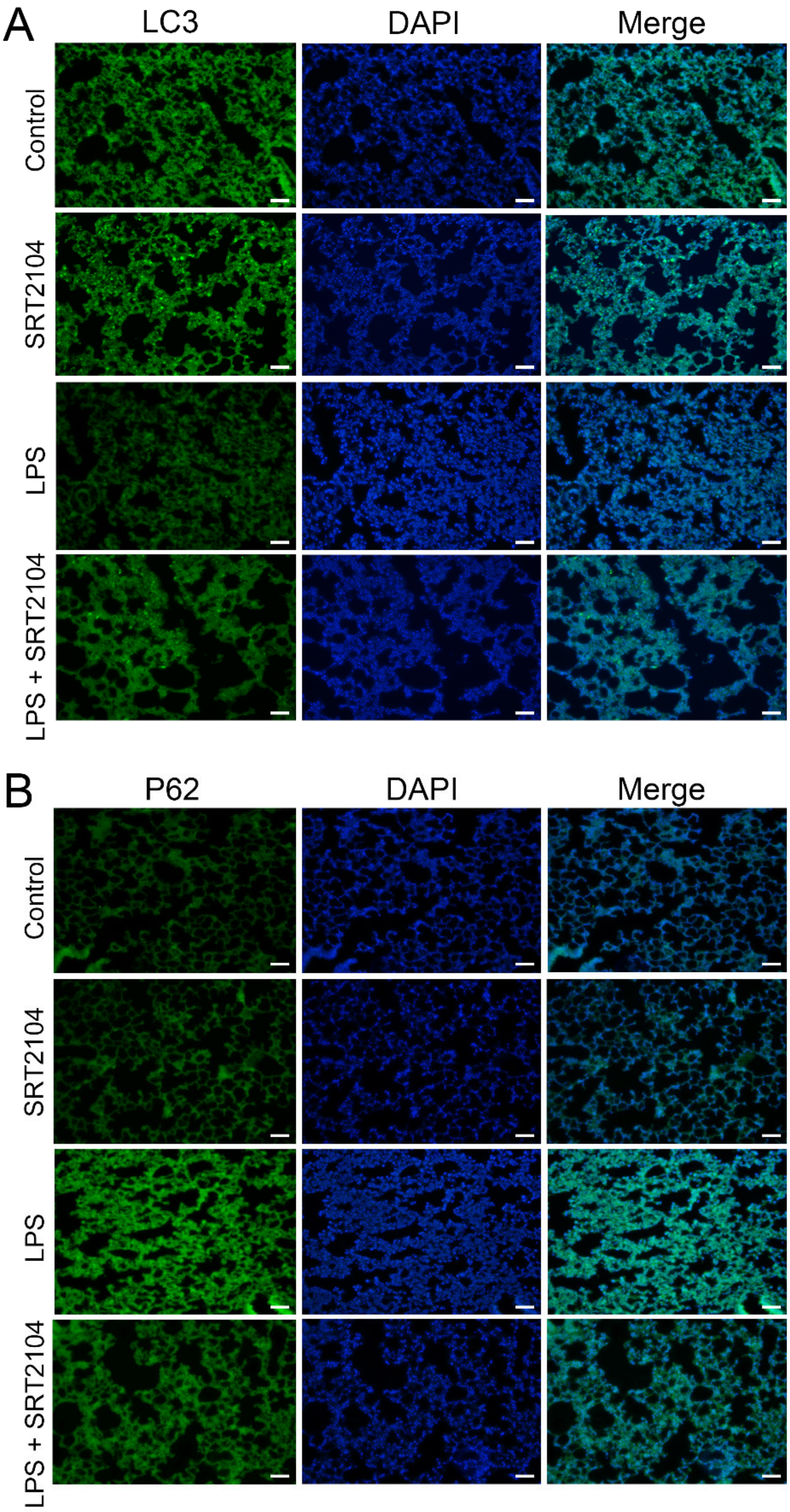

Supplementary figure 2. SRT2104 promoted autophagy in LPS-challenged mice. C57BL/6 mice were challenged with LPS to induce systemic inflammation, vehicle or SRT2104 (25 mg/kg) was administered intraperitoneally. The mice were sacrificed 8 h post LPS exposure. (A) LC3 and (B) p62 protein were visualized by immunofluorescence (Scale bar: 100 µm).

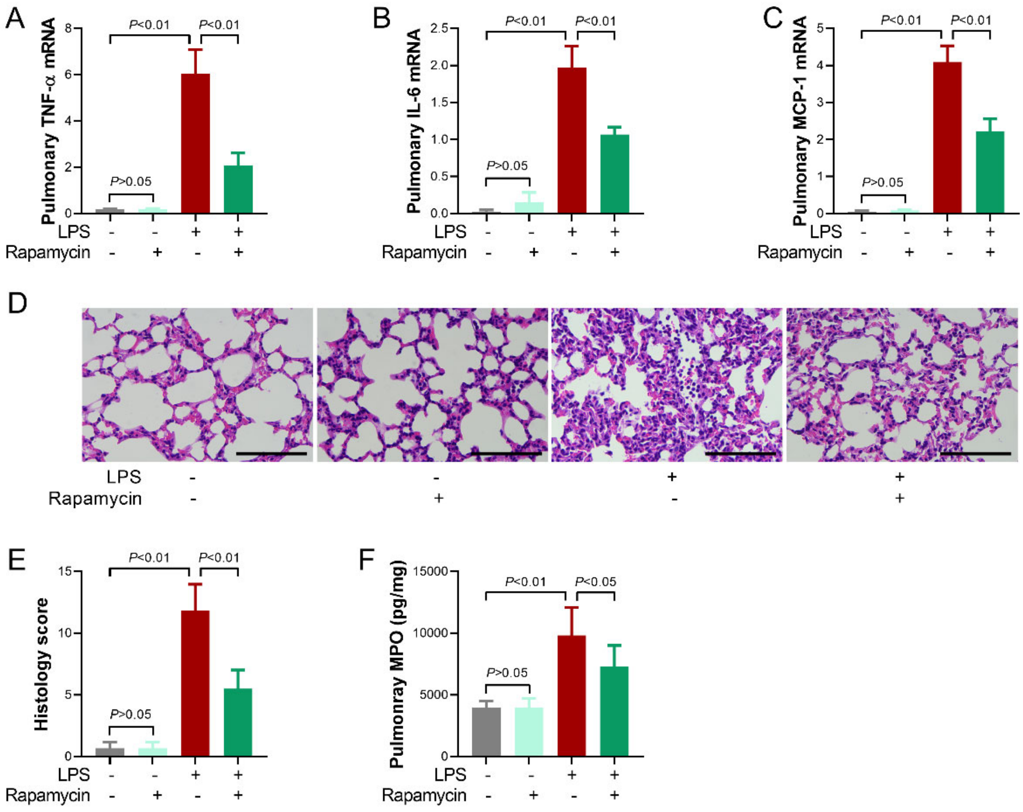

Supplementary figure 3. Rapamycin suppressed LPS-induced inflammatory lung injury. C57BL/6 mice were challenged with LPS to induce systemic inflammation, vehicle or rapamycin (5 mg/kg) was administered intraperitoneally. The mice were sacrificed 8 h post LPS exposure. (A - C) The mRNA levels of (A) TNF-α, (B) IL-6 and (C) MCP-1 in lung tissues were determined (n=4). (D) HE staining was performed in lung tissues and the representative image of each group was shown (Scale bar: 100 μm). (E) The histological score was calculated based on the HE-stained sections (n=8). (F) The level of MPO in lung tissue was determined (n=8). Data were expressed as means + SD.

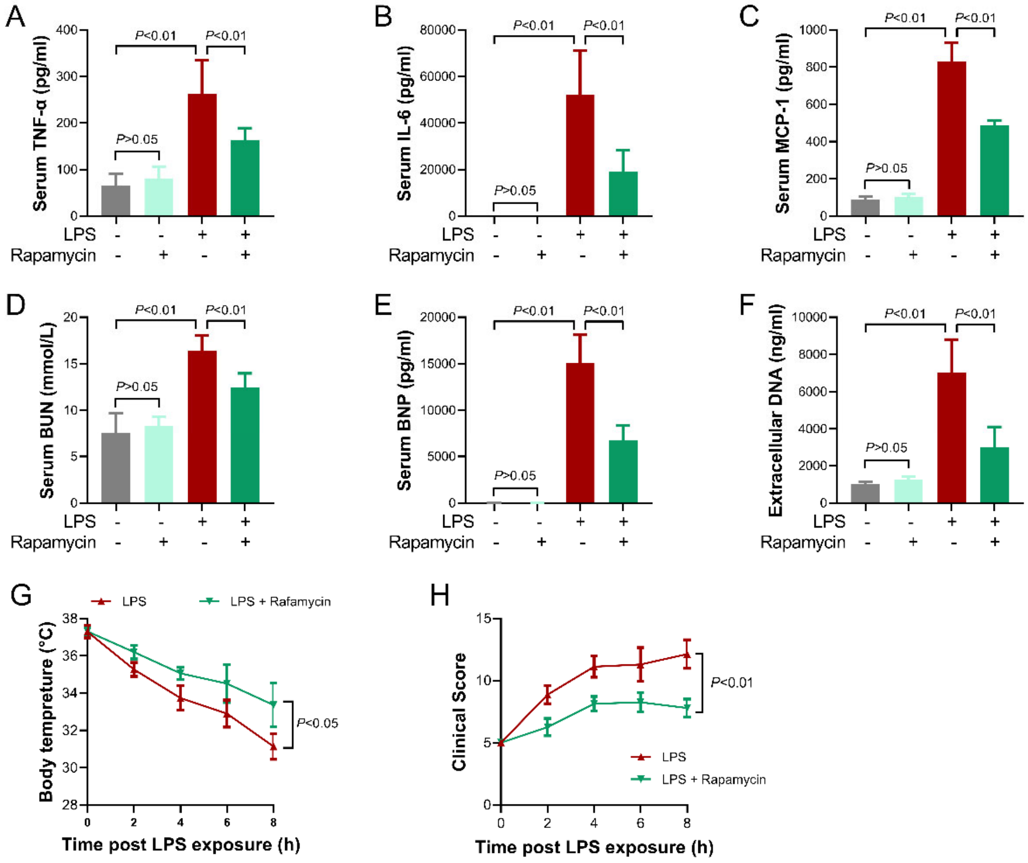

Supplementary figure 4. Rapamycin alleviated LPS-induced systemic inflammation. C57BL/6 mice were challenged with LPS to induce systemic inflammation, vehicle or rapamycin (5 mg/kg) was administered intraperitoneally. The mice were sacrificed 8 h post LPS exposure. (A - C) The serum levels of (A) TNF-α, (B) IL-6 and (C) MCP-1 were determined. (D and E) The serum levels of (D) BUN and (E) BNP were determined. (F) The level of extracellular DNA in serum was determined. (G) The body temperature of the experimental animals was recorded. (H) The clinical score of the experimental animals was calculated. Data were expressed as means + SD, n=8.
